# Supplementary material for: Steroidal Antimetabolites Protect Mice against Trypanosoma brucei
Source: Molecules. 2022 Jun 25;27(13):4088. doi: 10.3390/molecules27134088 (PMC9268410; doi:10.3390/molecules27134088)
Supplement: Supplementary file 1 [file molecules-27-04088-s001.zip › molecules-1753334-supplementary.pdf]

SUPPLEMENTARY MATERIALS

Steroidal Antimetabolites Protect Mice against *Trypanosoma brucei*

Minu Chaudhuri [1,*], Ujjal K. Singha [1,†], Boden H. Vanderloop [2], Anuj Tripathi [1] and W. David Nes. [2,*]

[1] Department of Microbiology, Immunology, and Physiology, Meharry Medical College, Nashville,
TN 37208, USA; ujjal.singha@vumc.org (U.K.S.); anuj1tripathi@yahoo.com (A.T.)

[2] Department of Chemistry & Biochemistry, Texas Tech University, Lubbock, TX 79409, USA; boden.h.vanderloop@vanderbilt.edu

* Correspondence: mchaudhuri@mmc.edu (M.C.); wdavid.nes@ttu.edu (W.D.N.)

† Present address: Department of Allergy, Pulmonary and Critical Care Medicine, Vanderbilt University Medical Center, Nashville, TN 37232, USA

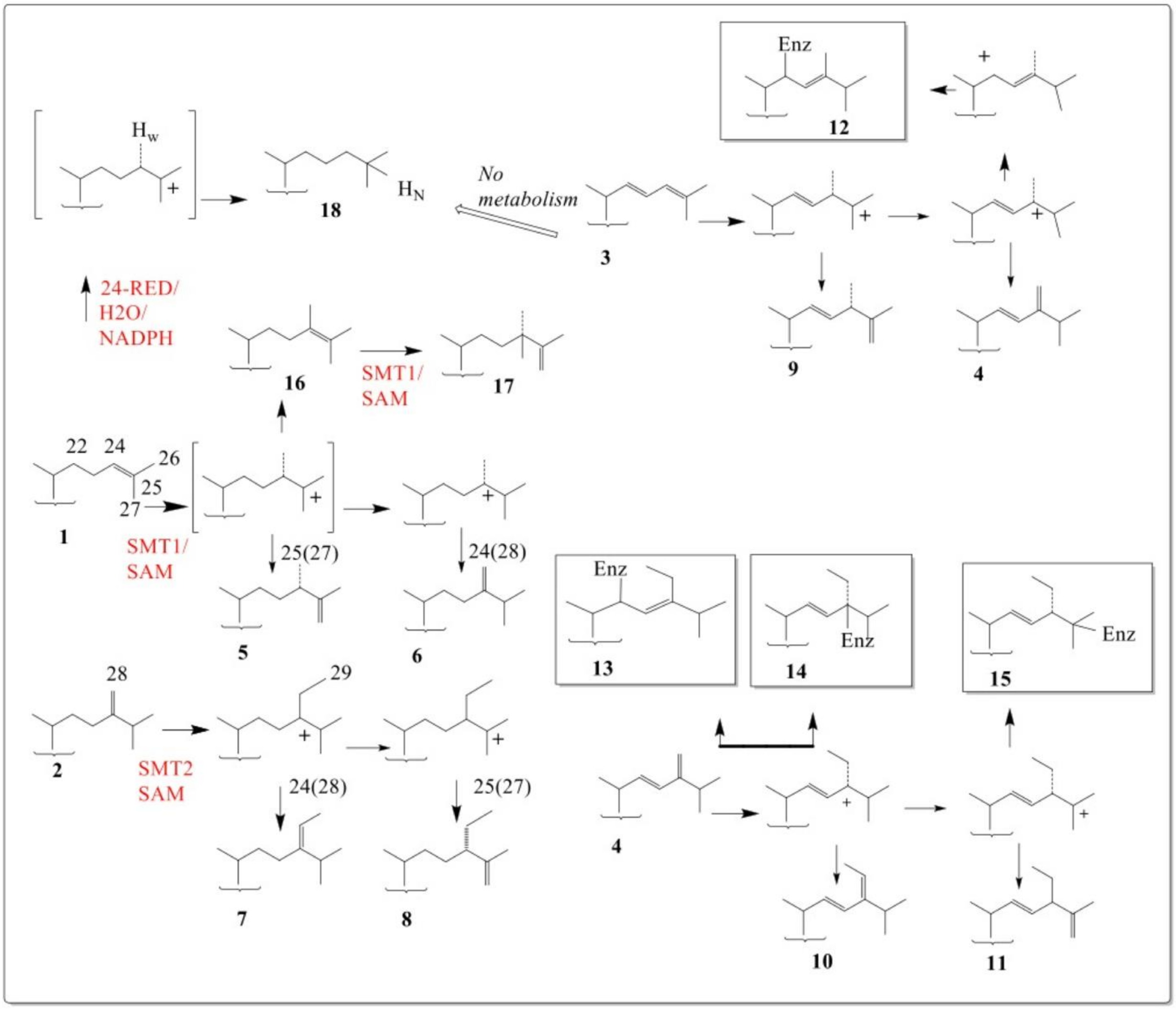

Figure S1. Sterol methylation steps catalyzed by SMT1 (first C24-methylation step) and SMT2 (second C24-methylation step) enzymes that occur across kingdoms after recognition of their natural or substrate analogue. Representative phyla-specific sterol C24/C28-methylation (**1** to **5/6/16/17** or **2** t **7/8**) and C24-reduction (**1** to **18**) (note: SMT = sterol methyltransferase enzyme, also indicated by Enz; SAM = *S*-adenosyl-L-methionine cofactor) pathways in the production of typical 24-alkyl sterols or the cholesterol side chain (note: the H_w = hydrogen/water versus H_N = hydrogen NADPH cofactor) from a $\Delta^{24(25)}$ sterol (**1**-such as zymosterol or cycloartenol—Figure 1) and interference by the suicide substrate-CHT and ERGT. The natural sterol methylation pathway ordto er differs somewhat across kingdoms. Thus, for the *Saccharomyces* yeast it is- **1, 2, 4 (CHT), 6** or in mutants **3** can convert to **4**; for soybean plants it is-**1, 6, 2, 7;** for the kinetoplastid *T. brucei* it is- **1, 5 or 1/16/17**; and for the *Acanthamoeba* parasite it is- **1, 6** or **1, 2, 8**. The sterol $\Delta^{24(28)}$–$\Delta^{25(27)}$ methylation bifurcation, as noted in the formation of **6** and **5** from **1** (performed by soybean SMT1 and *Ac* SMT1 or *Tb* SMT1, respectively) or **7** and **8** from **2** performed by soybean GmSMT2 or *Ac* SMT2, respectively), contribute to the specificity in suicide substrate design and effectiveness as an SMT inhibitor of these enzymes.

CHT (side chain structure **3**) or ERGT (side chain structure **4**) as drug supplements/antimetabolites incubated with *Ac* SMT or *Tb* SMT enzyme can convert in small measure to expected products of **4** and **9** or **10** and **11**, respectively, while aberrant reaction pathways (leading to inactivated enzyme boxed as for **12**, **13**, **14**, **15**) exist that produce aberrant outcomes of the intermediate binding covalently to the enzyme. Thus, for A. castellanii, the enzyme inactivation pathway is **3** to **12** or **4** to **13**, **14** and **15**. In addition, *T. brucei* has been found to operate the **3** to **12**. It is predicted that *T. brucei* SMT will also convert ERGT to a 24-alkyl carbocationic intermediate that binds covalently to the SMT because the second methyl transfer is reported to proceed via the $\Delta^{25(27)}$ -pathway yielding **17** (Zhou et al., 2006). Based on the HEK cell inability to metabolize the CHT side chain (while the cells could convert the CHT $\Delta^{(5,7)}$ system to Δ^5 we conclude the Δ^{24}-sterol reductase (24-RED) cannot metabolize Δ^{24}-bond when conjugated to the Δ^{22} (Zhou et al., 2019)

The use of SMT1 and SMT2 in the scheme correspond to their use in the literature. As we have reported in our studies cited in the references the substrate specificity for the different SMTs differ such that plants prefer cycloartenol (SMT1) or 24(28)-methylene lophenol (SMT2), yeast and *T. brucei* prefer zymosterol (SMT1), *Acanthamoeba* SMT1 and SMT2 prefer the plant-based substrates cycloartenol and 24(28)-methylenelophenol. Also relevant, yeast possess a single copy of SMT which only converts **1** to **6**- therefore it is considered SMT1. Soybean has three copies of SMT, two of which are responsible for the first methyl transfer (SMT1) while the other two are responsible for the second methyl transfer (one copy shown to be redundant) (SMT2). *Acanthamoeba* has two copies of SMT, one is responsible for the first methyl transfer (SMT1), the other is responsible for the second methyl transfer (SMT2). *T. brucei* has a single copy of SMT and therefore is considered a SMT1 yet it can weakly generate the unconventional second methyl group as in **17**. (Metabolic scheme was adapted from our previously published article [27]).
